# Disseminated tuberculosis complicated by hemophagocytic lymphohistiocytosis in an immunocompetent adult with favorable outcomes: A case report

**DOI:** 10.1016/j.idcr.2021.e01370

**Published:** 2021-12-22

**Authors:** Abdulrahman F. Al-Mashdali, Musaed S. Al Samawi

**Affiliations:** a Department of Internal Medicine, Hamad Medical Corporation, Doha, Qatar; b Department of Infectious Diseases, Hamad Medical Corporation, Doha, Qatar

**Keywords:** TB, HLH, Hemophagocytic syndrome, Disseminated tuberculosis, Infection, Case report

## Abstract

Hemophagocytic lymphohistiocytosis (HLH) is an uncommon hyperinflammatory syndrome characterized by excessive activation of macrophages and T-cells with high cytokines levels, causing multiorgan dysfunction.HLH has been associated with variable infectious etiologies, such as tuberculosis(TB). TB-associated HLH (TB-HLH) is a rare condition, but it is fatal if not treated. The diagnosis of TB-HLH is challenging and might be missed if not highly considered. The classic manifestations of HLH include pancytopenia, organomegaly, lymphadenopathy, and coagulopathy. Herein, we present a young immunocompetent adult diagnosed with disseminated TB complicated by HLH. Our patient responded well to the combination of antituberculosis therapy(ATT), corticosteroid, and intravenous immunoglobulin(IVIG). This case highlights the importance of considering this fatal complication in TB patients.

## Introduction

Disseminated tuberculosis (TB) is a life-threatening condition occurring due to the hematogenous spread of *Mycobacterium tuberculosis* (MTB)*.* The diagnosis is difficult because of its nonspecific clinical manifestations with multiorgan involvement [1]. The exact incidence of disseminated TB is not established, albeit, in a review, it is estimated to comprise< 2% of TB cases in immunocompetent adults [2]. Hemophagocytic lymphohistiocytosis (HLH) is a rare hyperinflammatory syndrome characterized by excessive activation of macrophage and T-cells, causing excessive cytokines production and subsequent immune-mediated injury of different body organs [3]. It usually occurs secondary to malignancy, infections, or autoimmune disorders [4]. HLH might rarely complicate the clinical course of disseminated TB. In a recent literature review (from 1975 to March 2014), only 63 cases of tuberculosis-associated HLH (TB-HLH) were found [5]. Herein, we report a challenging case of disseminated TB complicated by HLH in an immunocompetent adult with favorable outcomes. To the best of our knowledge, this is the first reported case in Qatar.

## Case presentation

A 36-year-old Indian female, previously healthy, presented to our emergency department with fever, and shortness of breath for five days. Upon further questioning, she also reported a history of fatigue, headache, and arthralgia. She denied night sweating, anorexia, or weight loss. Review of other systems was unremarkable. She was married and had two kids, and worked as a housemaid. She denied a recent history of travel (she had lived in Qatar for one year)or contact with sick people. On presentation, she was alert and oriented. Her vital signs were as follows: oral temperature of 39 °C, pulse rate of 128 beats/minute, blood pressure of 104/67 mmHg, respiratory rate of 34 breath/minute, and oxygen saturation of 88%. Her physical examination was remarkable for respiratory distress without abnormal breathing sounds. Cardiovascular, abdominal, and neurological examinations were unremarkable. She was admitted to the medical intensive care unit (MICU) because of the high oxygen requirement.

**Fig. 1:**
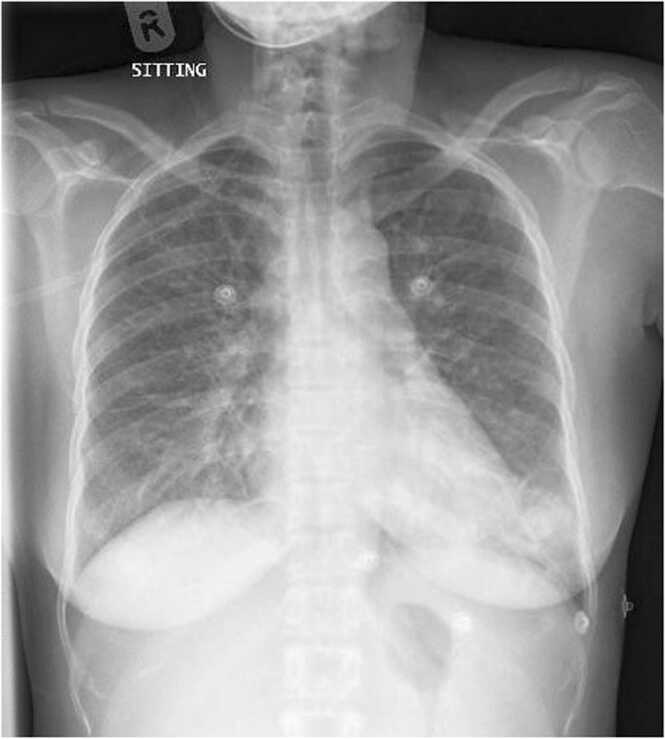
CXR showing scattered areas of air space opacities in left lower zone with minimal accentuated perihilar broncho-vascular markings.

Chest X-ray (CXR) showed scattered airspace opacities in the left lower zone (Fig. 1). Laboratory investigations were significant for pancytopenia, coagulopathy, elevated liver enzymes, and elevated inflammatory markers (Table 1). Abdominal ultrasound revealed minimal pleural effusion, minimal ascites, and moderate splenomegaly. COVID-19 polymerase chain reaction (PCR), respiratory viral panel, blood cultures, and human immunodeficiency virus(HIV) screen were all negative. In addition, pulmonary TB workup ( AFB sputum smear and PCR)and Quantiferon test were negative. The differential diagnosis was broad, including malignancy, autoimmune disease, and TB.

**Table 1:** Relevant laboratory results during hospital stay.

Parameter	Reference range	Day 1	Day 5	Day 10	Day 28 (one week after ATT)	Before discharge (after 3 months)
WBC	4–10 × 10^3/uL	0.9	1.5	2.2	3.5	3.1
Hemoglobin	12–15 gm/dL	7.8	9	7.7	7.5	9
Platelets	150–400 × 10^3/uL	81	63	45	43	148
INR	0.8–1.2	1.4	1.4	1.5	1.2	1.1
PT	10–12	13.5	14	15.1	13	11
APTT	30–34	36.5	37	42	32	29
Fibrinogen	1.7 − 4.2 gm/L	3.84	–	–	–	–
Creatinine	50–98 umol/L	32	49	55	64	80
Total Bilirubin	5–24 umol/L	12	43	52	256(mainly direct, drug induced?)	12
Albumin	35–52 gm/L	19	21	18	12	25
AST	0–30 U/L	160	280	155	224	28
ALT	0–30 U/L	63	101	87	56	22
Triglycerides	< 1.7 mmol/L	3.1	–	–	–	1.68
Ferritin	12–160 ug/L	1464	–	–	7668	1357
CRP	< 5 mg/L	82	–	191	108	22.5
Procalcitonin	< 0.5 ng/mL	1.07	–	66.2	1.1	–

Abbreviations: WBC: White blood cell; INR: International normalization ratio; PT: Prothrombin time; APTT: activated partial thromboplastin time; ALT: alanine aminotransferase; AST: aspartate aminotransferase; CRP: C-reactive protein

**Fig. 2:**
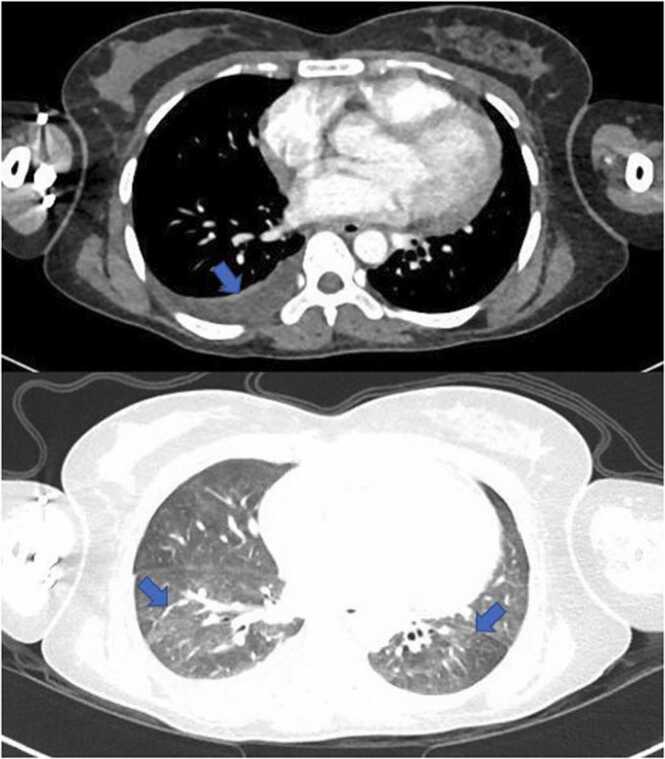
CT thorax showing right sided pleural effusion ( top), and bilateral ground-glass opacities mainly in the lower lung lobes(bottom).

**Fig. 3:**
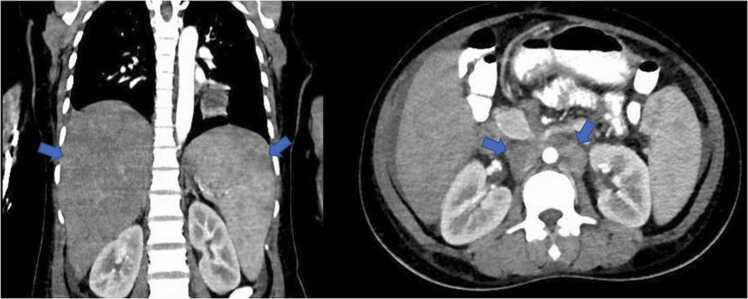
CT abdomen showing hepatosplenomegaly (left),and bulky enlargement of para-aortic lymph nodes (right).

We decided to request CT (computed tomography)of the thorax, abdomen, and pelvis to look for any focus or lymphadenopathy. Pan CT revealed minimal ascites and right pleural effusion, hepatosplenomegaly, multiple small non-enhancing lesions in the splenic parenchyma, and multiple enlarged para-aortic lymph nodes (Figs. 2 and 3). Empirical antibiotics(piperacillin-tazobactam followed by meropenem) were started to cover for sepsis of unknown origin. Bone marrow examination showed significantly increased marrow histiocytes noted with a prominent feature of hemo-erythrophagocytosis. On day 21 of the hospital stay, peritoneal fluid tapping was done and revealed positive MTB PCR. In addition, based on CT findings, a biopsy of the supraclavicular lymph node was done to rule out malignancy, specifically lymphoma.

Accordingly, the patient was treated as a case of disseminated TB with secondary HLH. She was started on antituberculosis treatment (ATT), isoniazid (INH), rifampin (RIF), ethambutol (EMB), and pyrazinamide (PZA). Also, she received treatment for HLH based on the 2004 Protocol (IVIG x 3 courses, dexamethasone, then prednisolone for a total of 6 weeks). The patient developed drug-induced hepatitis immediately after starting ATT, which was related to isoniazid (replaced by moxifloxacin). Later during hospital stay (after six weeks), sputum, peritoneal fluid, and supraclavicular lymph node cultures all grew MTB. The patient stayed in the hospital for almost three months. Upon discharge, her laboratory results are shown in Table 1. Because of the COVID-19 pandemic, the patient was following in TB virtual clinic. She showed significant improvement on the ATT regimen without any significant adverse effects. However, she traveled back to her home country in the fourth month of ATT, so we could not follow her until the end of therapy course.

## Discussion

HLH has been linked to a wide range of viral, bacterial, fungal, and parasitic infections. Epstein-Barr virus (EBV) is the most common infectious etiology associated with HLH. HIV has been associated with HLH; however, the HIV screen was negative in our patient. TB can lead to HLH on infrequent occasions [6]. The pathophysiology of HLH involves excessive immune system stimulation and cytokine storm. However, the underlying pathophysiology of TB-HLH is still unclear.MTB acts as an obligate intracellular organism after phagocytosis by macrophages. This activates macrophages and T-helper cells, further releasing a large number of cytokines and chemokines, resulting in TB -HLH clinical manifestations. In TB patients, the levels of interferon-g, tumor necrosis factor-a, and granulocyte/monocyte colony-stimulating factor are higher than those in a healthy population. On the other hand, HLH patients have increased levels of cytokines mentioned above (as a part of cytokines storm), which might explain how TB can lead to HLH [5], [7], [8].

HLH is a multisystem disease manifesting with different nonspecific clinical features. There are two forms of HLH: a primary (mainly genetic) form, mainly occurring in pediatrics, and a secondary (reactive) form, which is the most common form [7]. Malignancy accounts for up to 70% of adult cases and carries the worst prognosis [9]. The symptoms of TB-HLH are nonspecific and usually chronic, including fever, fatigue, anorexia, and weight loss. Most patients (65%) with TB-HLH have comorbid conditions; however, our patient did not have any comorbidity. The most common clinical signs of TB-HLH include fever(almost 100% of cases), hepatosplenomegaly(70% of cases), and lymphadenopathy(70% of cases). Regarding the laboratory findings in TB-HLH, pancytopenia has been detected in more than 50% of the reported cases. Interestingly, for unexplained mechanisms, serum triglyceride level has been found to be elevated in most TB-HLH cases, including our case [5]. There are two main diagnostic scoring systems for HLH: Hscore (the newest one)and HLH-2004 [3], [10]. The discussion of those diagnostic systems is beyond the scope of our case report, albeit our patient met the criteria for both diagnostic systems. Notably, the presence of hemophagocytosis on bone marrow aspirate is not essential to diagnose HLH ( sensitivity=83% and specificity=60%) [9]. Although MTB is present throughout the lungs in disseminated(or miliary) TB, acid-fast bacilli(AFB) could not be detected on sputum microscopy in several cases, as in this case. In our case, MTB was initially detected from PCR of peritoneal fluid, and then MTB was isolated from the cultures of different specimens (took up to 6 weeks) [11].

Treatment of TB should be started immediately in disseminated TB complicated by HLH. Regarding the immunosuppression use in TB-HLH, the corticosteroid is the cornerstone in the HLH 1994 and HLH-2004 treatment protocols. In the HLH-2004 protocol, etoposide, and cyclosporine A can also be used in HLH, however, a lower dosage is recommended in elderly patients due to their side effects. In addition, intravenous immunoglobulin (IVIG) and plasma exchange are effective in the treatment of severe HLH cases with multiple organ dysfunction syndromes (MODS) [3], [5], [8]. Our patient was treated successfully with the standard four-drugs (rifampin, isoniazid, pyrazinamide, and ethambutol) ATT, in addition to IVIG & corticosteroids ( based on HLH-2004 protocol). The overall prognosis of TB-HLH is unfavorable, with a mortality rate reaching 49% [5]. Several factors associated with a worse prognosis of HLH include malignancy, male gender, non-resolving fever, splenomegaly, coagulopathy,markedly elevated serum ferritin(>1000 ng/mL),and hypoalbuminemia [12]. Of note, all patients who did not receive any treatment for TB-HLH eventually died [5].

## Conclusion

TB might rarely be complicated by HLH, and the diagnosis is often challenging to the clinician. The clinician should consider HLH as an important differential diagnosis of TB patients with pancytopenia, organomegaly, and coagulopathy. Hscore and HLH-2004 are the main diagnostic scoring systems for HLH. Any delay in the definitive therapy will lead to increase morbidity and mortality. Corticosteroids and IVIG have shown to be effective in the treatment of severe TB-HLH. However, further studies are required to decide the optimal management for TB-HLH cases.

## Ethical approval

Ethical approval was obtained from Medical Research Center (MRC) in Hamad Medical Corporation (HMC).

## Funding

This research did not receive any specific grant from funding agencies in the public, commercial, or not-for-profit sectors.

## CRediT authorship contribution statement

**Abdulrahman F. Al-Mashdali:** Funding acquisition, Drafting the manuscript, Approval of the version of the manuscript to be published. **Musaed S. Al Samawi:** Funding acquisition, Revising the manuscript critically for important intellectual content, Approval of the version of the manuscript to be published.

## Consent

Written informed consent was obtained from the patient for publication of this case report and accompanying images. A copy of the written consent is available for review by the Editor-in-Chief of this journal on request.

## Declaration of Competing Interest

The authors have no conflict of interest to declare.
